# Endophytic bacterial dataset of the Cavendish banana grown in Dak Lak Province of Vietnam using 16S rRNA gene metabarcoding

**DOI:** 10.1016/j.dib.2023.109863

**Published:** 2023-12-07

**Authors:** Dinh Minh Tran, Thi Huyen Nguyen

**Affiliations:** Institute of Biotechnology and Environment, Tay Nguyen University, Buon Ma Thuot, Dak Lak 630000, Vietnam

**Keywords:** Endophytic bacteria, The cavendish banana, The dwarf cavendish cultivar, *Proteobacteria*, Biosynthesis

## Abstract

The Cavendish banana (*Musa cavendishii* L.) is one of the main perennial crops grown in Dak Lak Province of Vietnam. However, data on the endophytic bacterial community of this plant are unknown. In this work, a representative sample, mixing from 5 root samples collected from five banana gardens (the Dwarf Cavendish cultivar) in Dak Lak, was used for analyzing the endophytic microbiome using 16S rRNA gene metabarcoding. Results showed that 5 phyla, 7 classes, 20 orders, 31 families, and 47 genera of endophytic bacteria were identified from the sample. Bacteria belonging to phylum *Proteobacteria* were the most predominant, with 72.64%, and functions involved in biosynthesis were the most abundant, with 75.35%, of the endophytic bacterial community. Data help to understand the endophytic bacterial community of the Cavendish banana cultivated in Dak Lak, Vietnam. These data can be useful for further experiments concerning relationships between the growth of the Cavendish banana and endophytic bacteria. This is the first report on the endophytic bacteria of the Cavendish banana cultivated in Dak Lak, Vietnam.

Specifications Table

**Table:** 

Subject	Microbiology: Microbiome
Specific subject area	Molecular biology, Bioinformatics
Data format	Raw, Filtered, and Analyzed
Type of data	Figures, Fastq files
Data collection	A representative sample, mixing from 5 root samples collected from five Cavendish banana gardens in Dak Lak, Vietnam. The Swift Amplicon 16S plus ITS panel kit (Swift Biosciences, USA) was used to prepare the library. Metagenomic DNA of the root sample was extracted, and the Illumina MiSeq technology (2 × 150 PE) was used to sequence the library of the 16S rRNA gene (regions V1 to V9). The SILVA and PICRUSt2 databases were used to analyze the composition and functions of the endophytic microbiome, respectively.
Data source location	• Institution: Institute of Biotechnology and Environment, Tay Nguyen University • Province/Country: Dak Lak/ Vietnam • Latitude and longitude coordinates for collected samples: 12°32′40′′N, 107°59′39′′E; 12°32′38′′N, 107°59′32′′E; 12°32′31′′N, 107°59′34′′E; 12°32′30′′N, 107°59′38′′E; 12°32′47′′N, 107°59′56′′E
Data accessibility	Raw sequences (Fastq files) Repository name: Mendeley Data Data identification number: doi: 10.17632/y5shp3s365.2 Direct URL to data: https://data.mendeley.com/datasets/y5shp3s365/2

## 1 Value of the Data

• Data help to understand the taxonomic and functional profiles of the endophytic bacteria of the Cavendish banana cultivated in Dak Lak Province of Vietnam.
• Data can be useful for comparing the endophytic bacteria of the Cavendish banana and others.
• Data can be useful for further experiments concerning relationships between the growth of the Cavendish banana and the endophytic bacteria.

## 2 Background

Bananas are Vietnam's third largest fruit exporter, next to dragon and durian. According to a report, Vietnam planted 157,600 hectares and produced 2,514,800 tons of bananas in the year 2022 [1]. Bananas were grown everywhere in Vietnam, including Dak Lak Province. Among the banana cultivars, the Dwarf Cavendish is the primary cultivar grown in Dak Lak. To our knowledge, data on bacteria and their functional profiles in coffee, black pepper, sugarcane, and rice plants cultivated in this province have been reported [[2], [3], [4], [5]]; however, those of the Cavendish banana are unknown. This work aimed to establish a dataset on endophytic bacteria of banana (the Dwarf Cavendish cultivar) cultivated in Dak Lak, using 16S rRNA gene metabarcoding, for further experiments involved in relationships between the growth of the Cavendish banana and indigenously endophytic bacteria.

## 3 Data Description

**Fig. 1:**
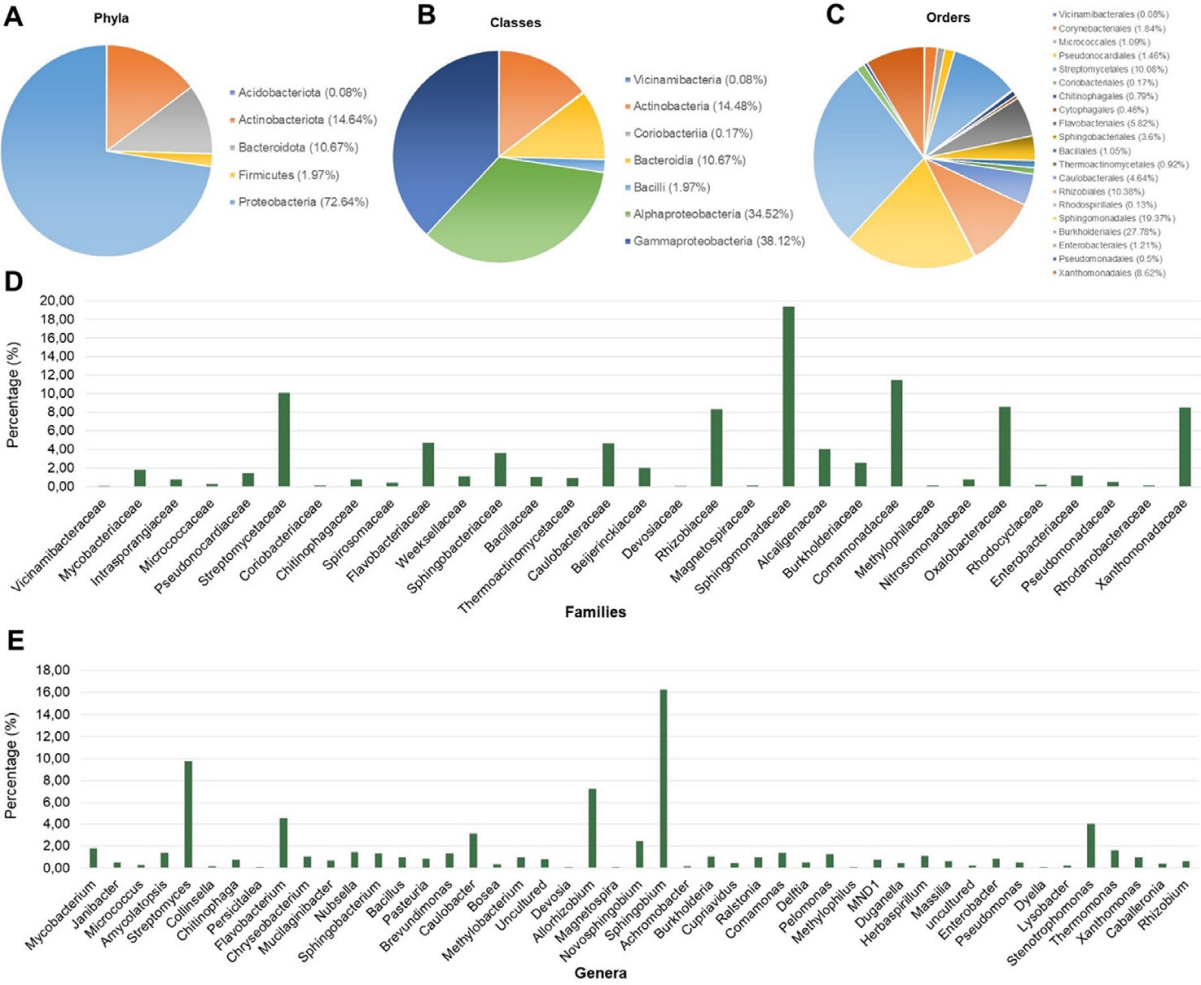
Taxonomic profiles of the endophytic bacteria of the Cavendish banana. *Note:* The taxonomic profiles of the endophytic bacteria at levels of phyla (A), classes (B), orders (C), families (D), and genera (E).

In this work, 125,933 reads were filtered from 293,974 raw reads and used for analysis. Results showed that 5 phyla, 7 classes, 20 orders, 31 families, and 47 genera of bacteria were identified in the representative sample. Among the phyla, *Proteobacteria* (72.64%) was the most predominant phylum, followed by *Actinobacteriota* (14.64%), *Bacteroidota* (10.67%), *Firmicutes* (1.97%), and *Acidobacteriota* (0.08%). Of the identified classes, *Gammaproteobacteria* (38.12%) was the primary class, followed by *Alphaproteobacteria* (34.52%), *Actinobacteria* (14.48%), *Bacteroidia* (10.67%), *Bacilli* (1.97%), *Coriobacteriia* (0.17%), and *Vicinamibacteria* (0.08%). Among the determined orders, *Burkholderiales* was the most abundant (27.78%), followed by *Sphingomonadales* (19.37%), *Rhizobiales* (10.38%), *Streptomycetales* (10.08%), *Xanthomonadales* (8.62%), *Flavobacteriales* (5.82%), and *Caulobacterales* (4.64%). Of the detected families, *Sphingomonadaceae* (19.37%) was the most abundant among these families, followed by *Comamonadaceae* (11.51%), *Streptomycetaceae* (10.08%), *Oxalobacteraceae* (8.58%), *Xanthomonadaceae* (8.49%), *Rhizobiaceae* (8.28%), *Flavobacteriaceae* (4.73%), *Caulobacteraceae* (4.64%), *Alcaligenaceae* (4.02%), and *Sphingobacteriaceae* (3.6%). Finally, *Sphingobium* was the primary genus (16.86%) of the identified genera (Fig. 1).

**Fig. 2:**
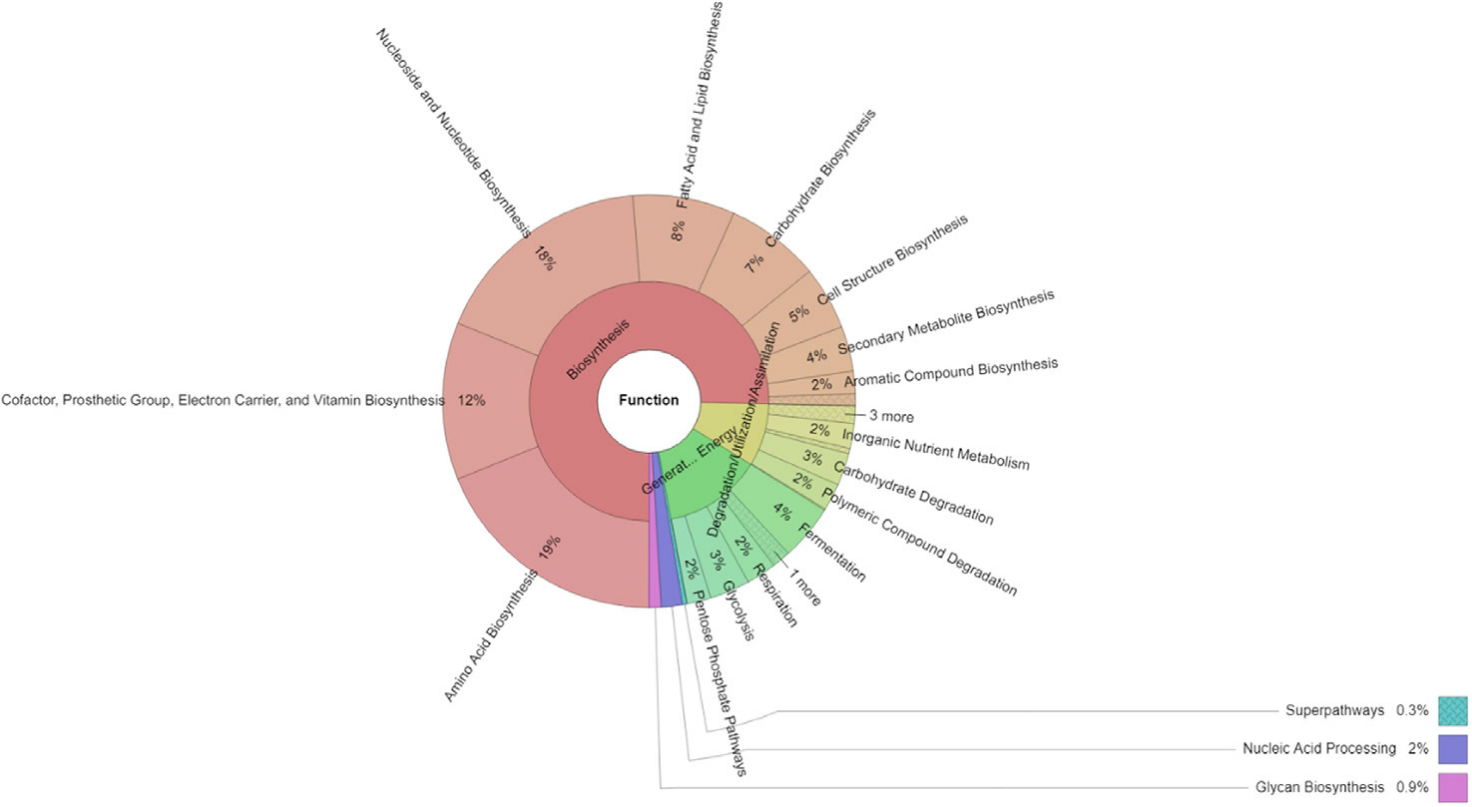
Functional profiles of the endophytic bacteria of the Cavendish banana.

Fig. 2 demonstrates that biosynthesis (75.35%) was the primary function of the Cavendish banana endophytic microbiome, followed by the generation of precursor metabolites and energy (13.26%), and degradation/utilization/assimilation (8.47%). Of the functions concerning biosynthesis, amino acid biosynthesis (18.86%) was the most abundant, followed by nucleoside and nucleotide biosynthesis (17.57%); cofactor, prosthetic group, electron carrier, and vitamin biosynthesis (12.31%); fatty acid and lipid biosynthesis (7.99%); carbohydrate biosynthesis (7.43%); cell structure biosynthesis (4.96%); and secondary metabolite biosynthesis (3.52%).

## 4 Experimental Design, Materials and Methods

### 4.1 Sample collection

Root sample collection, surface treatment, and maintenance were done as described previously [5]. Briefly, 5 root samples (about 50 g each) of banana, the Dwarf Cavendish cultivar, were collected from five gardens in Dak Lak, in the month of October in the year 2021. The samples were then combined to create a representative sample. The representative sample was kept at 4°C during the sampling, and the root surface was sterilized to remove microorganisms. The treated sample was stored at −80°C until the metagenomic DNA of the sample was extracted.

### 4.2 Genomic DNA extraction, library preparation, and sequencing

Genomic DNA extraction, library preparation, and sequencing were conducted as described previously [6]. Briefly, the DNeasy PowerSoil Pro kit (Qiagen, Germany) was used to isolate the metagenomic DNA from 300 mg of the root sample. Primers [7] were used to amplify the 16S rRNA gene (regions V1 to V9) of the metagenomic DNA, and the Swift amplicon 16S plus ITS panel kit (Swift Biosciences, USA) was used to prepare the library. The Illumina MiSeq platform (2 × 150 PE) was applied to sequence the library.

### 4.3 Analysis of data

Bioinformatic tools were used to analyze data as described previously [6]. Briefly, the Trimmomatic 0.39 and Cutadapt 2.10 were used to remove adapters, primers, and low-quality sequences. The q2-dada2 plugin and QIIME2 pipeline 2020.8 were used to cluster and dereplicate reads into amplicon sequence variants. The SILVA and PICRUSt2 2.3.0-b databases were used to predict taxonomic and functional profiles of the endophytic bacteria, respectively.

## Limitations

Not applicable.

## Ethics statement

The current work does not involve human subjects, animal experiments, or any data collected from social media platforms.

## CRediT authorship contribution statement

**Dinh Minh Tran:** Conceptualization, Methodology, Investigation, Formal analysis, Software, Data curation, Validation, Visualization, Writing – review & editing. **Thi Huyen Nguyen:** Investigation, Formal analysis.

## Data Availability

Endophytic bacterial dataset of the cavendish banana grown in Dak Lak, Vietnam (Original data) (Mendeley Data).
